# Effectiveness of an Educational Workshop on Palliative Care Knowledge in Lebanese Nurses

**DOI:** 10.1089/pmr.2023.0026

**Published:** 2023-10-30

**Authors:** Myrna A.A. Doumit, May Naifeh Khoury, Mary Arevian, Ghina Al Khatib, Suha Fares

**Affiliations:** ^1^ American University of Beirut, Hariri School of Nursing, Beirut, Lebanon.; ^2^ Clinical and Professional Development Center, American University of Beirut Medical Center, Beirut, Lebanon.

**Keywords:** education, end-of-life care, Lebanon, nursing, palliative care

## Abstract

**Background:**

Lebanon is one of the world's smallest countries, with an area of 10,452 square kilometers. Life expectancy in Lebanon presently stands at about 76.6 years for men and 79.3 years for women. It is well known that with long life comes chronic disease, serious illness, and increased resource utilization. With a rapidly aging population and ever-increasing life expectancy, an increase in illnesses that affect the elderly is expected to follow, including non-communicable diseases and cancer. Nurses are the largest workforce in Lebanon and are thus in a prominent position to influence the quality of palliative care (PC) delivery throughout the course of illness.

**Purpose:**

The purpose of this study was to evaluate the impact of an educational workshop on PC knowledge, attitude, and skills for practicing nurses at a Lebanese university medical center.

**Design:**

A mixed-method approach comprising a quasi-experimental and a qualitative process evaluation was followed to assess the nurses' knowledge, attitude, and skills about PC before and after the workshop and to evaluate the process itself. A convenience sample of 45 registered nurses working at the university medical center from multiple clinical units participated in the workshop that took place over one day in a referral medical center in Beirut. Inferential statistical analysis was used.

**Results:**

Data were analyzed using SPSS 25 for Windows. The paired *t* test showed a significant increase between the pre-and post-test scores *t* (39) = 11.07, *p* < 0.001 with a 95% confidence interval for the mean difference of (17.58–25.45). Thirty-eight participants (90.5%) did not pass the pre-test exam whereas only 12 participants (30.0%) did not pass the post-test exam.

**Recommendations:**

It is highly recommended to follow up with the participants of this workshop to determine the immediate and long-term outcomes of this educational workshop as well as offer workshops for a wider population of nurses in Lebanon and the region.

## Background

Lebanon is one of the world's smallest countries, with an area of 10,452 square kilometers. About 6.7 million people live in the country, with over 80% in urban areas.^1^ Lebanon's demographic statistics have evolved, with current life expectancy at birth increasing to 79 years.^2^ Lebanon is experiencing an epidemiological alteration, where diseases of wealth—diabetes, heart disease, cancer, high blood pressure—have markedly increased whereas diseases of poverty—infectious and communicable diseases—are declining but have not yet faded.^3^

The great burden of non-communicable diseases (NCDs) and the quickly aging population in Lebanon necessitates palliative care (PC) as an essential component of health services, which is indispensable to alleviate the distress of patients. Based on a report published in 2017, a projected number of 15,000 Lebanese patients require PC services each year.^4^ This number is anticipated to increase due to a number of factors such as the aging population, large Syrian refugee population (∼1.5 million over the past decade^5^), and the rise in NCDs.^4^

It is worth noting that in international, and mainly in low- and middle-income countries, a number of education and training models have been used to enable access to undergraduate and graduate training in PC nursing at generalist and specialists' levels.^6,7^

### Nursing and palliative care

The end of a person's life can be one of the most important times she/he experiences. While the way we die has changed considerably during the 21st century, Lebanese and most societies in the region still do not accept the idea of PC as they relate it to end-of-life care.^8^ Various studies conducted in Europe, the United Arab Emirates, and the Eastern Mediterranean Region highlight the need to have more training for advanced PC competences for physicians and nurses.^9–12^

In Lebanon, Abu Saad Huijer and Daher^13^ and Daher and Doumit,^14^ members of the National Committee for Pain Relief and Palliative Care (NCPRPC) identified the need for pain relief and PC as a priority. They emphasized undergraduate training of nurses and physicians on pain relief and PC and highlighted that PC content needs to be integrated in nursing and medical curricula. The authors emphasized the need for postgraduate training in pain relief and PC in the country.

Daher et al.^15^ stated that health professionals are increasingly interested in PC, and this interest is paralleled by increasing learning opportunities. However, <5% of those who deliver end-of-life (EOL) care that is under the PC umbrella have received undergraduate PC education. Most have acquired PC knowledge and skills after graduation.^15^ Daher et al.^16^ have also stressed that in order for PC services to become widely available, affordable, and socially acceptable, education of patients and professionals in PC is urgently needed in Lebanon.

Keeping in mind that if PC is done properly, it provides as good or better outcomes than curative care alone, is cost effective, and alleviates pain and suffering for patients and caregivers alike.^16,17^ In a Knowledge to Policy (K2P; analysis of context, problem, policy options and implementation barriers) policy brief by Soueidan et al.^4^ on integrating PC into the health system in Lebanon, strengthening education and training in PC among health professionals were amid the main points listed.

Despite stepped-up efforts to educate nurses on PC and EOL care, significant barriers and challenges continue to exist in providing adequate educational resources to nurses actively engaged in delivering or supporting PC and EOL care. In fact, in the Atlas of PC in the Eastern Mediterranean region, it was noted that in 22 responding countries no nursing schools were reported to have an independent nursing course in PC.^17^

In the Middle East, the growth of PC is still in early stages despite all efforts in multiple Arab countries.^12,18^ PC programs in the region are hospital-based, either as hospital-based consultation services or as dedicated inpatient units. Saudi Arabia, Jordan, and Egypt have the highest number of PC programs. No PC service providers have been found in Syria, Libya, Yemen, Djibouti, or Somalia.^17^

Some of the challenges of PC that have been consistently addressed in the literature state that PC is not sufficiently integrated in nursing and medical curricula in the region, and even in affluent countries, most caregivers have received little or no PC training.^17^ A recent study^11^ in the United Arab Emirates revealed a lack of structured PC training and limited provision of formal teaching and assessment of trainee knowledge and skills in palliative and EOL care.

Nursing educators and leaders in Lebanon and international nursing leaders indicate a collective awareness of the need to have efforts put together to include concepts of PC and EOL care in the curricula of the professional nurses and to prepare nurses to cope with death and dying as a potential outcome for patients under their care.^3,19–21^

In Lebanon, an educational effort toward training health professionals in PC is in progress. In 2007, the World Health Organization (WHO) pioneered a Public Health Strategy (PHS) for integrating PC into Lebanon's health care system through the education of policy makers, health care workers, and the public.

The goal was to implement both national PC programs and national cancer control programs, wherein PC was one of the four key pillars of comprehensive cancer control: (1) appropriate polices, (2) adequate drug availability, (3) education of health care workers, and (4) education of the public. This approach provides an effective strategy for integrating/establishing PC into a country's health care system.^22^

In 2008, May Naifeh Khoury, a faculty member of the School of Nursing at the American University of Beirut, recommended a model for future outlook that advocates for the need to continue to include concepts of PC and EOL care in the curricula of the professional nurses in a systematic way, through the development of a Foundation Course in Palliative and EOL Care.^23^ It is only through offering a course in Palliative and EOL Care that nurses can make an impact on the quality care of their patients and families.^24^

Fourteen years later, one can argue that there have been some opportunities and attempts for change, which led to recognized progress in relation to the field of PC worldwide and in Lebanon.^4,25^ In line with the complete PHS in Lebanon, the Ministry of Public Health's aforementioned NCPRPC^26^ has begun work toward implementing PC in Lebanon through a national plan to deliver change in pain relief and PC across the life span and for various illnesses. The focus of this committee is on four broad areas: education, practice, research, and public policy.

With respect to PC education, a subcommittee was created with the purpose of developing a national curriculum, training health care providers in principles of PC, and promoting public awareness through integrating PC in health education across Lebanon. Consequently, a modular curriculum, a one-credit course on PC for undergraduate medical and nursing students, was recommended and for practicing health professionals as continuing education workshops.

However, to date, in Lebanon, there are no follow-up/evaluative published studies of the effectiveness of those educational endeavors. Similarly, a few evaluations of the effectiveness of educational and training programs are published in nursing literature in the Middle East region.^27^ A review of PC publications from the Eastern Mediterranean Region between 2005 and 2016 identified 73 unique articles, which denotes the scarcity in funding and output in PC in the region.^17^

A failure to strategically provide Lebanese nurses with PC skills, education, and supportive policies will certainly lead to unmitigated serious health-related suffering and an increased economic burden on the national health system.

It is worth noting that the definition of PC embraced in this workshop is the one adopted by the Lebanese committee for pain and PC and it reads as follows: “Palliative care is an approach that improves the quality of life of patients and their families facing the problems associated with life-threatening illness, through the prevention and relief of suffering by means of early identification and impeccable assessment, and treatment of pain and other problems—physical, psychosocial and spiritual.”^28^ The definition of PC comprises the end-of-life care as well.

In summary, there is a clear need to develop and evaluate educational interventions designed to improve the ability of practicing health professionals in PC, in Lebanon, as well as in the region. The purpose of this study was to evaluate the impact of an educational workshop on PC knowledge, attitude, and skills for practicing nurses at a Lebanese university medical center and recommend future educational programs.

## Methods

### Design and sample

A mixed-method approach comprising a quasi-experimental and a qualitative process evaluation were followed to meet the purpose of this study. An invitation to nurses working on medical-surgical adult, critical care areas and other areas such as multiple sclerosis, post-anesthesia, and respiratory care unit was extended to attend the workshop through the nursing office as part of a process improvement plan that the nursing office adopted.

A convenience sample of 45 registered nurses working at the university medical center from multiple clinical units responded and were selected by the nursing office to participate in the workshop in 2019.

Inclusion criteria:

1. Registered nurses working on the medical- surgical, critical care areas and other areas (other areas include multiple sclerosis, post-anesthesia, and respiratory units)
2. Registered nurses working for >6 months

Of the 45 registered nurses who attended the workshop, 42 took the pre-test and of those, 39 completed the post-test.

### Setting

The workshop took place in Lebanon over one day (10 hours including breaks) in a building annexed to the medical center during working hours of the nurses. The environment was conducive to learning, as it contained all equipment needed to facilitate a smooth transfer of information between the audience and the experts.

### Development and implementation

A day-long educational workshop on the Basics in PC was developed and implemented by a group of local expert physicians and nurses in PC. The content of the workshop was developed based on the principles of the Adult education theory.

The objectives of the workshop were to: (1) Enhance nurses' knowledge of PC; (2) evaluate types of pain and accordingly implement pain management modalities; (3) critically analyze gaps, elements, models and key concepts of PC; (4) identify causes and management of physical and psycho-social symptoms in PC; (5) develop skills in communicating “bad news” to patients and care givers; and (6) develop skills in meeting patients' last hour needs.

Teaching/learning strategies included lectures with PowerPoint presentations; discussion sessions with audio-visual aids; open questioning; and reflections on videos.

Local PC experts, both physicians and nurses, provided the lectures. The topics chosen for the lectures were adapted from the Education in Palliative and End-of-Life Care (EPEC) curriculum. The lecture series included: History and Evolution of Palliative Care; Principles of Palliative Care; Palliative Care in Lebanon; Pain assessment and pharmacological pain management; Symptom Assessment and Management; Psychological Dimensions of Palliative Care; Communication of Bad News; Ethical and Legal Issues; Goals of Care and Advance Care planning; and Care of Patients in their last hours of life and those who are bereaved.

### Data collection

Each participant nurse completed a self-administered pre- and post-test provided by an administrative assistant not connected to the study. Demographic data were collected to describe the study sample. Thirty-four multiple-choice questions prepared by the experts from their individual presentations were used to evaluate the knowledge, attitudes, and skills of the participants at the beginning and the end of the workshop.

Twelve questions were related to knowledge, 11 to attitude, and 11 to skills. Questions were developed based on bloom's taxonomy. Content and face validity of the questions were checked by two experts with academic background. It took 30 minutes to complete the questions. All questions were multiple-choice questions. The pre-test was administered before the workshop and the post-test an hour after completion of the workshop.

The questionnaire was graded out of a total of 100%. All questions had the same weight. An explanation was given about the techniques on how to answer the questions. Answers were recorded on an answer sheet, anonymously. Answer sheets were labeled as pre- or post-tests, collected in separate envelopes, and then taken for computer analysis. In addition to the objective test, a qualitative/process evaluation was conducted by the local expert group to evaluate the overall content and organization of the educational offering. Participants were asked as well to give feedback about the content and flow of the lectures, duration of the workshop, and level of difficulty of the material.

### Analysis

Inferential statistical analysis was used. Data were analyzed using the SPSS 25 for Windows. Total test scores before and after attending the sessions were summarized by means, standard deviations (SD), and range and compared using the paired *t* test. A two-tailed test was used, and a *p*-value <0.05 was considered significant. The process evaluation was analyzed and summarized by two experts to reflect on the participants' perceptions of the program.

### Ethical considerations

Considering that this study is part of a process improvement at the Medical Center, no Institutional Review Board (IRB) was required but legal approvals were sought from administration. Confidentiality of participants and data were secured.

## Results

The majority of the participants were females (77.8%), which parallels the distribution of nurses in Lebanon. Overall, 88.9% were between 20 and 30 years; 11.1% were between 30 and 40 years; 22.2% have 1–5 years of experience; 66.7% have 5–10 years of experience; and 11.1% have >10 years of experience. Those figures mirror the demographic distribution of the nursing workforce in Lebanon.

The distribution of the participants according to working unit was as follows: 44.4% worked in the oncology units, 33.1% worked on medical surgical units, 13.3% on critical care units, and 11.1% from the multiple sclerosis and post-anesthesia units. Overall, 88.9% of the nurses were BSN holders and 11.1% had a graduate degree (Table 1).

**Table 1.** Demographic Characteristics of the Attending Nurses at the Palliative Care Workshop in Lebanon

	Count	%
Gender (female)	35	77.8
Unit		
Oncology	20	44.4
Medical surgical	14	31.1
Critical	6	13.3
Other^a^	5	11.1
Age (years)		
20–30	40	88.9
30–40	5	11.1
Years of experience		
1–5	10	22.2
5–10	30	66.7
More than 10	5	11.1

^a^ Other include multiple sclerosis, post-anesthesia care unit, and respiratory care unit.

Thirty-nine participants completed both the pre- and post-test with a mean pre-test score of 49.30 (SD = 8.95) and post-test score of 70.54 (SD = 12.62). The demographics of those who did not complete the post-test do not differ much from their colleagues who sat for the pre- and post-test. The minimum score on the pre-test was 29 and the maximum was 65 whereas on the post-test the minimum and maximum scores were 47 and 88, respectively.

The paired *t* test showed a significant increase between the pre-and post-test scores *t* (36) = 11.07, *p* < 0.001 with a 95% confidence interval for the mean difference of (17.58–25.45). Thirty-eight participants (90.5%) did not pass the pre-test exam, whereas only 12 participants (30.0%) did not pass the post-test exam (Table 2).

**Table 2.** Difference Between the Pre-Test and Post-Test Scores (*N* = 39)

Outcome	Pre		Post		***p***	Mean difference post-test–pre-test	95% CI, mean difference
	Mean	SD	Mean	SD			
Total test score	49.30	8.95	70.54	12.62	<0.001	21.24	17.58–25.45

CI, confidence interval; SD, standard deviation.

Results revealed that during the pre-test, the three content areas: knowledge, attitude, and skills were problematic for the participants. Post-test improvements were remarkable in the knowledge and skills content; however, the attitude concept was still problematic. Moreover, the pain management and communicating bad news sections are still areas that need follow-up. This might be due to cultural factors related to the stigma of receiving opioids and discussing bad news with patients and family members by nurses in Lebanon.

Results of the process evaluation indicated that all the participants were satisfied with the content of the workshop; they found it very beneficial for their nursing practice.

The participants indicated that they had learned a lot, including information about communication skills, pain management, and support of the care givers. Their positive feedback indicated that the program constituted a good learning experience for them. However, they expressed concern that the material was too much to be covered in one day and recommended to offer the workshop over two days.

## Discussion

This is the first study to evaluate the effectiveness of a PC educational workshop on nurses' knowledge and attitude at a university medical center in Lebanon. Although this initiative on PC educational intervention for nurses was a first attempt, results indicated positive findings in knowledge and skills for PC care among the participants; however, the attitude of Lebanese nurses toward PC remains an area of concern.

Similar findings except for the concept “attitude” were reported in a large study^29^ conducted in China with >10,000 nurses. An online survey compared the PC knowledge and attitude scores of the matched pairs of respondents for the 17% who had taken a Jiangsu Nursing Association PC training program.

The results demonstrated that those who had received the training had statistically significant higher PC knowledge and better attitudes toward dying patients, although not in knowledge about psychosocial and spiritual care or attitudes toward the need to include families in patient support. A recent pilot study about nurses' knowledge in PC in North Lebanon emphasized that promoting continuous education is needed among nurses.^30^

Literature from multiple cultures also validates that specific training fosters professional nurses' knowledge of palliative and EOL care. In a U.S. study^31^ of 73 nurses (including more than one-third from oncology), given one 40-minute lecture, results reported a statistically significant improvement in the nurses' post-test scores.

In an oncology unit in the United States, researchers measured the knowledge, attitudes, and behavior of 46 oncology nurses at baseline and one month after participating in a four-hour class modified version of the EOL Nursing Education Consortium (ELNEC) curriculum for oncology nurses.^23^ One month after taking the course, the nurses completed the post-test and recorded the number of conversations they had with patients and families about PC before and after participating in the class.

Statistically significant increases were seen for knowledge, attitudes, and behaviors about PC as measured by a modified version of the Scale of EOL Care in the Intensive Care Unit (EOLC-ICU). Behaviors measured by the number of PC conversations showed a 20% rise in the number of nurses engaging in three or more conversations.

O'Shea and Mager^32^ explored the outcome of a six-week (12 classroom hours) ELNEC course with a group of 134 nurses' knowledge and attitude on working with patients who were dying with a pre- and post-test evaluation. The authors found a statistically significant improvement in knowledge and attitude (less negative) in post-test results.

In Jordan, a recent study^33^ investigated the impact of an e-learning PC curriculum from the International Children's Palliative Care Network on 120 pediatric nurses across two hospitals (one hospital served as a control and the other as the intervention group). The authors found a statistically significant increase in knowledge and attitudes toward PC for the intervention group, but not for the control group using a pre-test/post-test design.

A five-year PC education program for physicians, nurses, and other health care professionals was conducted across the state of Maharashtra, India, in three model sites.^34^ The program consisted of multiple activities and a training manual with teaching modules delivered twice a year. A questionnaire to evaluate the project was created and conducted by telephone with one quarter of the participants. More than 80% of the nurses responding mentioned they were now knowledgeable about PC and their practice for addressing pain and symptoms had improved.

The results of all these studies endorse the results of our study excluding the attitude component.^23,31–34^ It is evident that changing the attitude for such a delicate topic requires more time and training.

It is clear that even short courses, or courses taught online are effective in improving PC knowledge, attitudes, and behaviors in health care workers, nurses in particular in high-income countries as well as lower-middle-income countries such as Lebanon. Educational PC courses and training for all nurses working in PC units in Lebanon will foster their competence in this key component of health care delivery.

It is well known that the generation of evidence in one country does not always translate to another; therefore, it is imperative for nursing schools in Lebanon and nursing departments at leading medical centers to strategically plan to integrate PC into local curricula and continuing education programs respectively. This endeavor challenges educational institutions in Lebanon to offer further continuing education aimed toward updating and renewing the competence and expertise of professionals working within PC.

As PC competence is crucial for nurses worldwide, comprehensive, competency-based education will be needed to prepare them for practice with palliative patients and their significant others. Results also support the recommendations of the NCPPC in Lebanon for integrating PC into nursing and medical curricula and for developing continuing education sessions for working health professionals.

It is important to mention that health care workers cannot practice what they do not know. Therefore, the integration of such knowledge in the continuing education plan or academic curricula will make an impact on the quality of care, the patient's satisfaction, and ultimately nurses' satisfaction, which would lead to better retention.

### Strengths

This study, as a first attempt, has several strengths. It adds to the limited body of knowledge in the area of evaluating impact of PC education on health professionals, specifically nurses in the Middle East. Our findings also show the interdisciplinary partnership between Lebanese nurses and physicians in PC education. This study also demonstrates that even a one-day workshop can have a significant impact on nurses' knowledge and skills toward PC, thus highlighting the great need and setting the stage for continuing education. It also highlights the importance of working more on the attitude of nurses toward PC.

### Limitations

One limitation was that the questionnaire was not validated. However, the questions were based on current PC literature identified by the expert lecturers in the area. Another limitation was participant selection criteria and number of participants, a convenience sample, since the selection was done by the nursing office based on nurses' response to the call.

## Conclusion and Nursing Implications

Nurses in Lebanon are the largest workforce and are thus in a prominent position to influence the quality of PC delivery throughout the course of illness. All nurses should receive training on PC and continuing education. This study demonstrated that a tailored workshop on PC can make a difference in nurses' knowledge and skills. More work needs to be done to have an impact on the attitude of Lebanese nurses toward PC.

It is highly recommended to revise the content of this workshop and to check the validity and reliability of the questionnaire used. Also, there should be a follow-up on the participants of this workshop to determine the immediate and long-term outcomes of this educational workshop as well as offer workshops for a wider population of nurses in Lebanon and the region. We hope that the results of the study would feed into bigger studies in Lebanon and the region.

## Abbreviations Used

EOL: end of life
ELNEC: EOL Nursing Education Consortium
EOLC-ICU: EOL Care in the Intensive Care Unit
ICPCN: International Children's Palliative Care Network
K2P: Knowledge to Policy
LMICs: low- and middle-income countries
NCDs: non-communicable diseases
NCPRPC: National Committee for Pain Relief and Palliative Care
PC: Palliative Care
PHS: Public Health Strategy
SD: standard deviations
WHO: World Health Organization
